# Real‐time 3D motion estimation from undersampled MRI using multi‐resolution neural networks

**DOI:** 10.1002/mp.15217

**Published:** 2021-10-26

**Authors:** Maarten L. Terpstra, Matteo Maspero, Tom Bruijnen, Joost J.C. Verhoeff, Jan J.W. Lagendijk, Cornelis A.T. van den Berg

**Affiliations:** ^1^ Department of Radiotherapy University Medical Center Utrecht Utrecht The Netherlands; ^2^ Computational Imaging Group for MR Diagnostics & Therapy Center for Image Sciences University Medical Center Utrecht Utrecht The Netherlands

**Keywords:** deep learning, artificial intelligence, MRI, radiotherapy, registration, motion estimation, MRI‐guided radiotherapy, adaptive radiotherapy, MR‐Linac

## Abstract

**Purpose**: To enable real‐time adaptive magnetic resonance imaging–guided radiotherapy (MRIgRT) by obtaining time‐resolved three‐dimensional (3D) deformation vector fields (DVFs) with high spatiotemporal resolution and low latency (<500 ms).

**Theory and Methods**: Respiratory‐resolved $T_{1}$‐weighted 4D‐MRI of 27 patients with lung cancer were acquired using a golden‐angle radial stack‐of‐stars readout. A multiresolution convolutional neural network (CNN) called TEMPEST was trained on up to 32× retrospectively undersampled MRI of 17 patients, reconstructed with a nonuniform fast Fourier transform, to learn optical flow DVFs. TEMPEST was validated using 4D respiratory‐resolved MRI, a digital phantom, and a physical motion phantom. The time‐resolved motion estimation was evaluated in‐vivo using two volunteer scans, acquired on a hybrid MR‐scanner with integrated linear accelerator. Finally, we evaluated the model robustness on a publicly‐available four‐dimensional computed tomography (4D‐CT) dataset.

**Results**: TEMPEST produced accurate DVFs on respiratory‐resolved MRI at 20‐fold acceleration, with the average end‐point‐error <2 mm, both on respiratory‐sorted MRI and on a digital phantom. TEMPEST estimated accurate time‐resolved DVFs on MRI of a motion phantom, with an error <2 mm at 28× undersampling. On two volunteer scans, TEMPEST accurately estimated motion compared to the self‐navigation signal using 50 spokes per dynamic (366× undersampling). At this undersampling factor, DVFs were estimated within 200 ms, including MRI acquisition. On fully sampled CT data, we achieved a target registration error of 1.87±1.65 mm without retraining the model.

**Conclusion**: A CNN trained on undersampled MRI produced accurate 3D DVFs with high spatiotemporal resolution for MRIgRT.

## 1 INTRODUCTION

Real‐time adaptive radiotherapy aims to increase the accuracy with which radiation is delivered, leading to increased sparing of healthy tissue and organs‐at‐risk (OARs).^1^ By rapidly acquiring images and estimating tumor motion during dose delivery, the radiation beam can be adapted to follow the current anatomy. To facilitate treatment adaptation, magnetic resonance imaging–guided radiotherapy (MRIgRT) is increasingly adopted in clinical practice, for example, with the introduction of hybrid MR‐Linac devices.^2^, ^3^, ^4^, ^5^, ^6^ With its superior soft‐tissue contrast, MRI facilitates direct visualization of tumors and OARs.^7^, ^8^

For *real‐time* treatment adaptation, image acquisition and motion estimation must occur with low latency and a high spatiotemporal resolution,^9^ that is, the maximum time between a (respiratory) motion event and dose delivery should be ≤500 ms.^10^ However, real‐time acquisition of three‐dimensional (3D) MRI and computation of a nonrigid deformation vector field (DVF) is challenging due to the long acquisition times of fully sampled MRI (seconds to minutes) and the ill‐posed and underdetermined nature of motion estimation, hindering real‐time motion estimation.^11^, ^12^

Several methods have been presented to accelerate MR acquisition and motion estimation, such as parallel imaging,^13^, ^14^, ^15^ simultaneous multislice acquisitions,^16^ advanced image reconstruction algorithms allowing for greater undersampling factors, such as compressed sensing,^17^ or novel motion estimation methods model from 2D MRI.^18^ Recent works proposed using low‐rank models to reconstruct highly undersampled MRI with subsecond temporal resolution,^19^ but these methods currently have long reconstruction times. Currently, none of these methods can achieve the required acceleration factor combined with low‐latency reconstruction to estimate motion within 500 ms.^10^

Recently, deep learning (DL) has been proposed to speed up MRI reconstruction and motion estimation, achieving performances on par, if not higher, than its non‐DL counterparts.^20^, ^21^, ^22^, ^23^, ^24^, ^25^ Specifically, DL models allow for fast inference, leaving the time‐consuming step to the training phase, which can take hours or days.

In a previous work, we introduced a supervised DL‐based framework for real‐time 2D motion estimation.^26^ By reconstructing highly undersampled golden‐angle radial acquisitions with a nonuniform fast Fourier transform (NUFFT), motion was estimated by a multiresolution convolutional neural network (CNN), allowing for fast and accurate motion estimation.

Ideally, we could extend this approach to real‐time 3D motion estimation by training a 3D network on 3D cine‐MRI acquired with high spatiotemporal resolution. However, it is challenging to obtain high‐quality ground‐truth DVFs from in‐vivo MRI acquired at a high spatiotemporal resolution as the images will suffer from severe artifacts due to undersampling and respiratory motion. One way to circumvent this problem is by performing *respiratory‐sorted* image reconstruction instead of *time‐resolved* image reconstruction. Respiratory‐sorted MRI displays physiological motion similar to time‐resolved MRI, maintaining higher image quality as fewer motion artifacts due to less severe undersampling.

In this work, we extend the previously introduced 2D approach to 3D by training a DL model named TEMPEST (real‐**t**ime 3D motion **e**stimation fro**m** undersam**p**l**e**d MRI using multire**s**olution neural ne**t**works) to estimate DVFs from highly accelerated 3D‐MRI. We train TEMPEST on respiratory‐sorted 4D‐MRI to learn ground‐truth DVFs computed using conventional registration methods. The trained network is subsequently used to estimate motion from highly accelerated time‐resolved MRI. We investigate the optimal model hyperparameters and evaluate the model performance on digital and physical phantoms and 4D respiratory‐resolved CT data. Moreover, we estimate the performance of TEMPEST on time‐resolved MRI of two healthy volunteers acquired on an MR‐Linac.

## 2 METHODS

We trained a supervised multiresolution DL model (TEMPEST) to estimate a DVF (${\text{DVF}}_{\text{TEMPEST}}$) between two undersampled MRI volumes acquired with a golden‐angle radial stack‐of‐stars readout. The model requires MRI for training, together with ground‐truth DVFs (${\text{DVF}}_{\text{GT}}$) describing the motion between a dynamic and static volume.^1^

### 2.1 Patient data collection and preparation

Twenty‐seven patients undergoing radiotherapy for lung cancer between February 2019 and February 2020 at the RT department were retrospectively included under the approval of the local medical ethical committee with protocol number 20‐519/C.

Free‐breathing 3D golden‐angle radial stack‐of‐stars (GA‐SOS) $T_{1}$‐weighted spoiled gradient echo MRI of the thorax were acquired for 7 min on a 1.5T MRI (MR‐RT Philips Healthcare, Best, the Netherlands) during gadolinium injection (Gadovist, 0.1 ml/kg). The acquisition was fat‐suppressed using Spectral Attenuated Inversion Recovery (SPAIR). The relevant scan parameters are listed in Table 1 (4D MRI).

**TABLE 1:** Relevant scan parameters for three experiments. The “4D MRI” column describes the MR parameters for the respiratory‐resolved 4D‐MRI used for training, validation, and testing. The “Phantom experiments” columns refer to the two experiments acquiring a stationary and moving motion phantom on an MR‐linac. The “Time‐resolved MRI experiments” columns refer to MRI data acquired on an MR‐linac of two healthy volunteers for evaluation of real‐time motion estimation performance

		Phantom experiments		Time‐resolved MR‐Linac experiments	
Parameter	4D MRI	Stationary phantom	Moving phantom	Volunteer 1	Volunteer 2
Readout	GA‐SOS	GA‐SOS	GA‐SOS	GA‐SOS	Kooshball
Number of coils	28	8	8	8	8
TR/TE (ms)	3.2/1.3	4.3/1.8	3.4/1.5	3.4/1.5	3.5/1.4
Flip angle (${}^{\circ }$)	8	10	10	10	10
Bandwidth (Hz/px)	866	866	868	865	868
FOV (mm${}^{3}$)	440×440×270	440×440×270	440×440×270	525×525×270	525×525×525
Resolution (mm${}^{3}$)	2.13×2.13×3.50	2.0×2.0×3.5	4.9×4.9×3.5	5.0×5.0×3.5	4.9×4.9×4.9
Matrix size	206×206×77	220×220×77	90×90×77	106×106×77	108×108×108
Slice direction	FH	FH	FH	FH	‐
Scan time (s)	438	60	28	163	40

Patients were scanned in supine position using a 16‐channel anterior and 12‐channel posterior phased‐array coil. In total, 1312 radial spokes per slice were acquired, corresponding to approximately four times oversampling compared to a fully sampled volume, which requires 206·π/2≈324 spokes. To train and evaluate the motion estimation model, patients were divided in a train set (17 patients), validation set (five patients) to find optimal hyperparameters and prevent overfitting, and a test set (five patients) to evaluate the final model performance.

### 2.2 Image reconstruction

**FIGURE 1:**
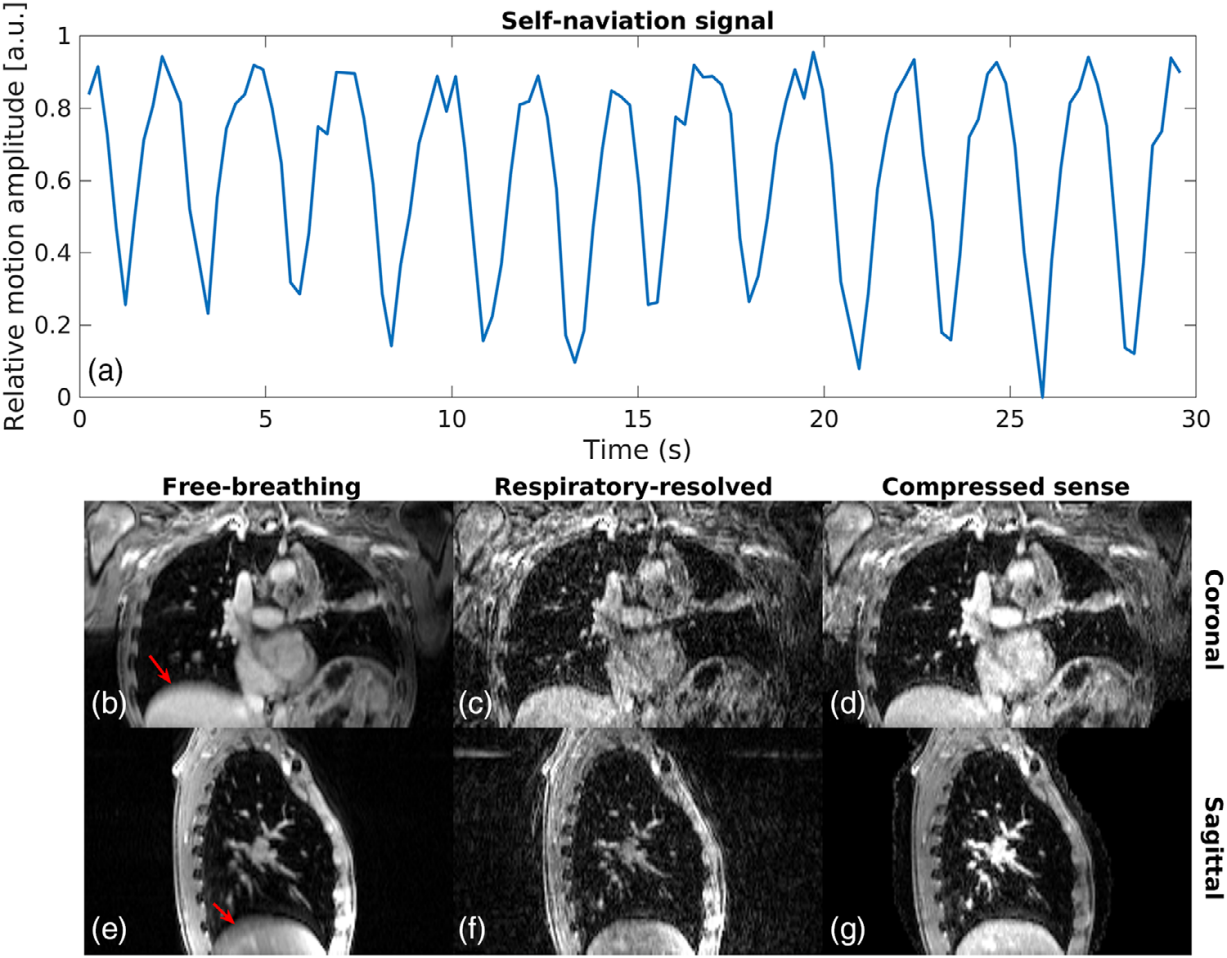
Examples of the self‐navigation signal and the data. A typical example of the self‐navigation signal during the first 30 s of the acquisition (a). In a free‐breathing reconstruction (b, e), blurring due to motion can be observed near the red arrows. With a respiratory‐resolved NUFFT reconstruction (c, f), the blurring is resolved at the cost of undersampling artifacts. Compressed‐sense reconstructions (d, g) show improved image quality at the cost of longer reconstruction times

To train TEMPEST with physiological motion, we reconstructed respiratory‐resolved MRI based on the self‐navigation signal present in the 4D‐MRI,^27^ as illustrated in Figure 1(a). An example of respiratory‐resolved reconstruction versus free‐breathing image reconstruction is shown in Figures 1(b), (c), (e), and (f). The self‐navigation signal was obtained by sampling radial spokes and performing a 1D Fourier transform of center of k‐space, that is, $k_{0}$, along the slice direction. The respiratory motion surrogate was obtained by performing principal component analysis on the concatenated navigators.^28^, ^29^

As contrast was injected, the relative magnitude of the self‐navigation signal changed over time. To account for the contrast wash‐in phase, we discarded the first 200 spokes of every scan to prevent contrast mixing. The remaining spokes were sorted based on the respiratory phase and relative amplitude using a hybrid binning algorithm.^30^ After sorting, k‐space was density‐compensated using a Ram–Lak filter, interpolated onto a twice‐oversampled Cartesian grid using a 3×3 Kaiser–Bessel kernel, and transformed to image‐space with an NUFFT‐adjoint reconstruction^31^, ^32^ with a weighted coil combination. Four‐dimensional respiratory‐resolved magnitude reconstructions were made for 1, 3, 5, 10, 20, 30, 40, 50, 75, and 100 respiratory phases. As 1312 spokes were sampled in total, and 324 sampled spokes are required to fulfill the Nyquist criterion, the undersampling factor of the respiratory‐resolved MRI is computed as $R=(324\cdot n_{\text{phases}})/1312$, corresponding to approximately 0.25‐, 0.75‐, 1‐, 3‐, 5‐, 10‐, 13,‐ 15‐, 18‐, and 27‐fold undersampling, respectively. As we aimed to train a multiresolution motion estimation model, we also reconstructed images at a lower spatial resolution, that is, 2× and 4× spatial downsampling, by radially cropping the k‐space around $k_{0}$, reducing the spatial resolution in the left–right and anterior–posterior direction. Along the feet–head (Cartesian) direction, resolution was maintained. The reconstructed images were normalized by scaling the image intensity to an output range of [0, 1] by clipping to the 99th percentile of the image intensity. The percentiles were computed on a patient basis over all respiratory phases.

To validate TEMPEST at high undersampling factors, that is, R=10, 13, 15, 18, 27, we required ground‐truth DVFs for comparison. However, traditional methods were unable to provide accurate DVFs based on the adjoint reconstructed images due to the undersampling artifacts. Therefore, MRI was also reconstructed using compressed sense with temporal total variation (TV) regularization, λ=0.03.^17^, ^29^ An example of these reconstructions is shown in Figures 1(d) and (g).

### 2.3 Ground‐truth motion

Ground‐truth DVFs were computed using optical flow,^33^, ^34^ as it provided a good balance between computation time, registration performance, and number of hyperparameters. Optical flow computes motion by assuming spatial smoothness of the DVF, regularized by the β hyperparameter. A preliminary study, which is presented in Supporting Information Figure S4, was performed to select the optimal value for β=0.4 for our training data.

**FIGURE 2:**
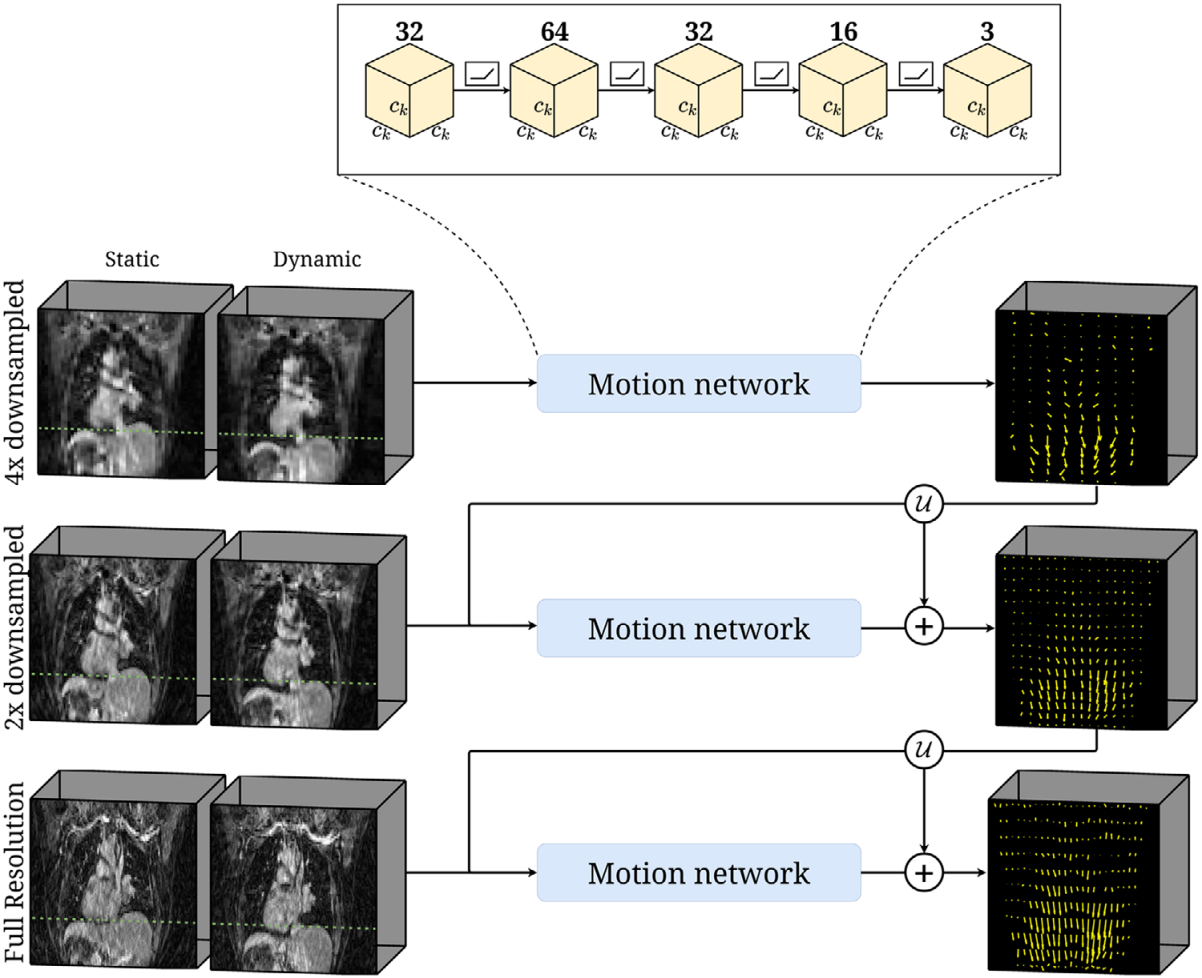
Model overview. TEMPEST computes the DVF between a static and dynamic volume, where the green line indicates the motion magnitude. TEMPEST starts at 4× spatially downsampled resolution. A motion network, consisting of a 3D CNN of five layers (32, 64, 32, 16, and 3 learned filters, respectively), which operates on the whole volume, estimates the DVF between a static and a dynamic volume. This first motion estimate is upsampled through U and serves as an additional input for the motion network operating at the next resolution level. The subsequent layers learn the residual DVF that improves the previous estimate. The size of all convolution kernels is $c_{k}\times c_{k}\times c_{k}$, depending on the resolution level. All layers but the last are followed by an ReLU nonlinear activation

**FIGURE 3:**
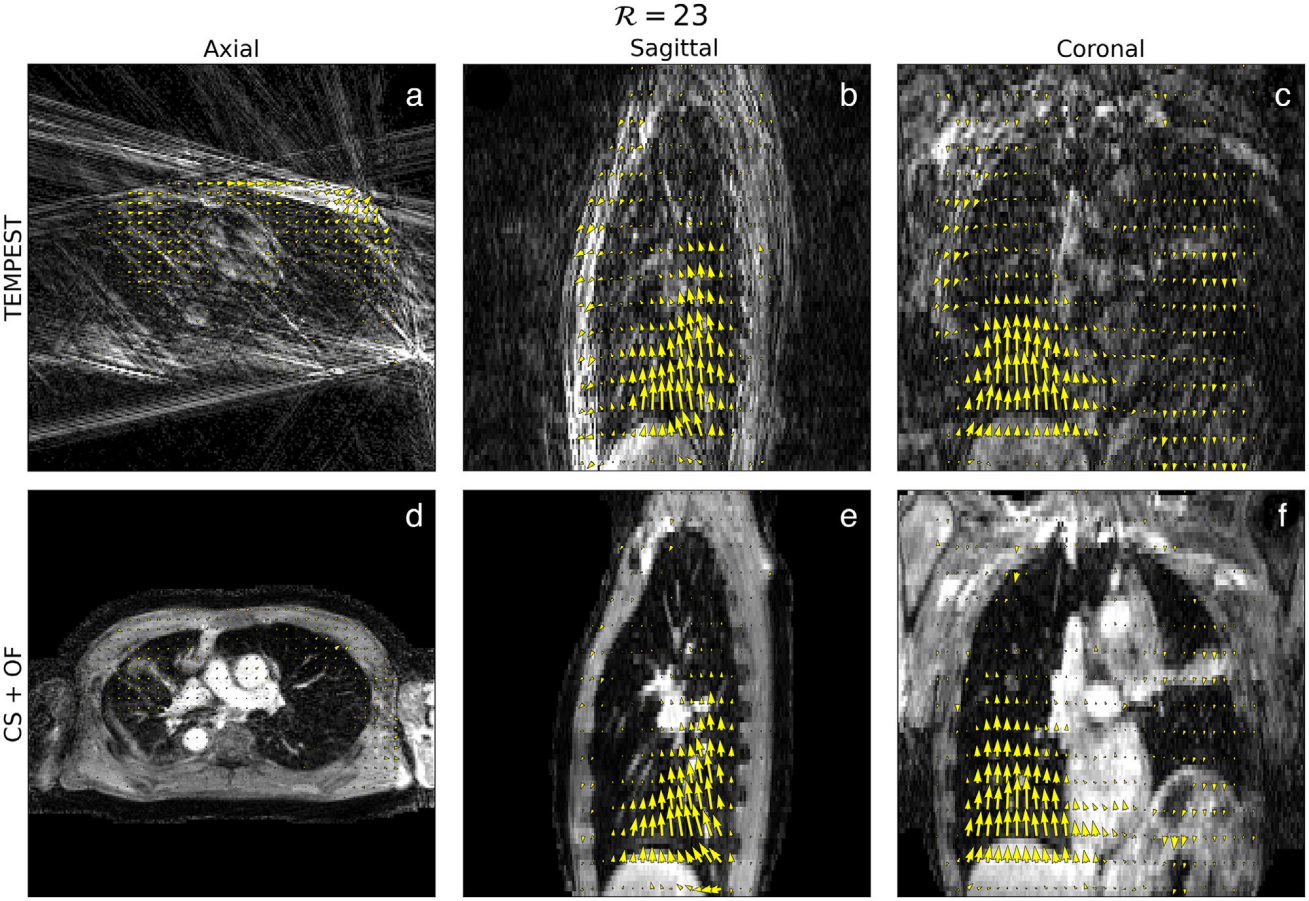
Example of motion estimation. An example of motion estimation in 3D by TEMPEST (a–c) on 23‐fold undersampled MRI. Good correspondence can be observed between the motion estimated by TEMPEST and motion estimated by optical flow computed on compressed sense reconstructions (d–f). In the quasi‐static region, TEMPEST estimates slightly larger residual motion. In the Supporting Information Videos S1–3, animated figures are provided

**FIGURE 4:**
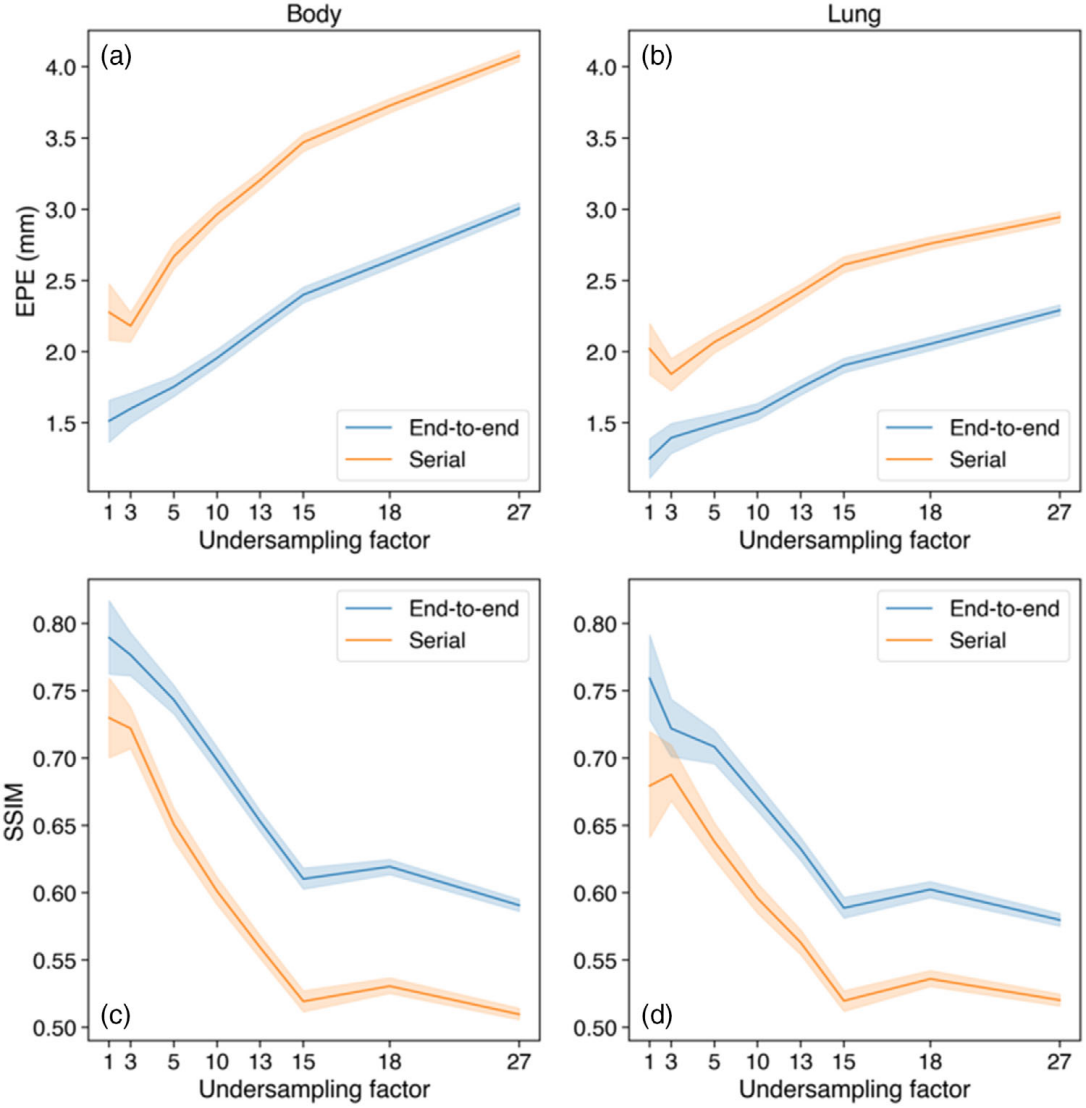
Comparison of serial versus end‐to‐end training on 4D MRI. Two variants of TEMPEST were trained: with end‐to‐end backpropagation (blue) and serial training (orange). EPE (a, b) and the SSIM of registered compressed sense reconstructions (c, d) as a function of the undersampling factor. Evaluation was done within the body contour (a, c) and within the lungs (b, d)

We calculated optical flow DVFs (${\text{DVF}}_{\text{OF}}$) for each respiratory‐resolved dynamic to three static volumes: full inhale, full exhale, and halfway inhale and exhale. This increased the training data and to ensure that the network learned to compute motion in multiple principal directions.

Optical flow was computed up to 20 respiratory phases (i.e., R≈7) at full resolution. For R>10, optical flow DVFs (${\text{DVF}}_{\text{CS,OF}}$) were computed on the compressed sense reconstructions as the motion estimate became unreliable due to the artifacts present in the undersampled NUFFT‐adjoint reconstructed MRI.

### 2.4 Network architecture

TEMPEST was designed as a multiresolution 3D convolutional neural network (CNN) operating on the entire volume to learn the DVF between a static and dynamic image. The complete motion model consisted of K multiresolution motion networks, operating on different spatial resolution levels. Each motion network had a fixed architecture and consisted of five 3D convolution layers with 32, 64, 32, 16, and 3 filters of size $c_{k}\times c_{k}\times c_{k}$, respectively. The motion network that operated at the lowest resolution directly attempted to learn a DVF from a static and dynamic volume. The motion network that operated at higher resolution levels received a static volume, dynamic volume, and upsampled DVF from the previous motion network as input, and attempted to learn a residual DVF to refine the upsampled DVF from the previous motion network. Several works perform intermediate warping of the dynamic images according to the estimated DVF.^35^, ^36^, ^37^ In a previous work,^26^ we identified that warping undersampled images using the estimated DVF was detrimental to the motion estimation performance. As performing 3D image interpolation is a costly operation, we opted to omit this operation. Each convolution layer in a motion network, except for the final layer, was followed by an ReLU nonlinear activation function.^38^ Figure 2 depicts the model architecture. TEMPEST was trained to minimize the end‐point‐error $\left(\text{EPE}=||{\text{DVF}}_{\text{GT}}-{\text{DVF}}_{\text{TEMPEST}}|{|}_{2}\right)$ by considering the magnitude error and angle error as separated terms and penalizing nonsmooth DVFs. The full loss function that was minimized during training was

$$L=\alpha \cdot \left(L_{\text{mag}}+L_{\phi }\right)+\left(1-\alpha \right)\cdot L_{\text{EPE}}+\lambda \cdot \nabla \text{DVF,}$$

where $L_{\text{mag}}$ is the $\ell_{2}$‐norm of the magnitude difference between the target and output DVF, $L_{\phi }$ is the $\ell_{2}$‐norm of the difference in angle between the target and output DVF, $L_{\text{EPE}}$ is the EPE that is, the $\ell_{2}$‐norm of the difference of the output DVF and target DVF, and ∇DVF enforces smoothness of the DVF by penalizing the mean Laplacian of the DVF. For our experiments, we used $\lambda =10^{-5}$.

The motion networks were trained sequentially, starting at the lowest resolution level. When the validation loss of this network converged, the motion network operating at the next higher resolution level was trained. At that point, two training strategies were considered for training the next motion networks: conventional “serial” training and “end‐to‐end” training. During serial training, no backpropagation was performed over the low‐resolution motion networks when training the higher resolution motion networks. With end‐to‐end‐training, however, backpropagation *was* performed over the lower resolution levels. We investigated this scheme based on the idea that it allows the low‐resolution network to learn features that are more expressive for high‐resolution motion estimation than the DVF at that level.

The final performance depended partially on the model hyperparameters. Good hyperparameters were found through a representative grid search, searching among the following values:

- α: The weight factor between the EPE and variable‐split terms, α∈[0.0,0.1,0.2,…,1.0].
- K: The number of resolution levels to use, K∈[3,4].
- $c_{k}$: The sizes of the convolution kernels in the convolution kernels for every resolution level k∈K, $c_{k}\in \left[3,5,7\right]$.

This resulted in a total of 1188 different model configurations, which were trained on five patients and evaluated on three patients. For each of the 1188 combination of hyperparameters, a model was trained for 50 epochs on five patients with a fixed random seed. We selected the hyperparameters corresponding to the model that achieved the lowest average EPE on three unseen patients. With these hyperparameters, we trained TEMPEST with serial and end‐to‐end training strategies on the training set of 17 patients. Both models were identically initialized and trained deterministically to prevent unintended advantages on the train set of 17 patients. Respiratory‐resolved MRI was made for every patient in the train set with multiple undersampling factors. In total, the train set consisted of 2108 static/dynamic/DVFs samples with undersampling factors R∈[1,3,5,7].

To prevent overfitting, the model performance on the validation set was evaluated after every epoch. The models were trained using the Adam optimizer with a base learning rate of $lr=10^{-4}$ and with $10^{-3}$ $\ell_{2}$ weight decay on a GPU (Tesla V100, NVIDIA, Santa Clara, CA, USA) with 32GB VRAM. We also used a learning rate schedule that halved the learning rate if the average validation loss did not decrease with at least $\Delta L=10^{-8}$ during 10 epochs.

Both models were trained until convergence of the validation loss was observed, that is, the average validation loss did not decrease more than $\Delta L=10^{-8}$ during 10 epochs and the learning rate was smaller than $10^{-8}$. During training, we performed augmentation on the static and dynamic MRI and the DVFs using TorchIO^39^ by performing random flips along an axis (p=0.5), applying a random bias field (p=0.25, order ∈[0,1,3,5]), and adding random Gaussian noise to the volumes (p=0.25, μ=0, σ∼U(0,0.05)). After initial training, the full model was fine‐tuned for 100 epochs on a dataset consisting for 25% of image pairs from the training set up to R=7 *with* motion (i.e., nonzero ground‐truth DVF), and for 75% of image pairs of the training set between 7‐ and 32‐fold undersampling *without* motion (i.e., the ground‐truth DVF is zero everywhere) to decrease sensitivity to undersampling artifacts.

To increase inference speed, the fully trained models were quantized from full‐precision (fp32) to half‐precision (fp16) after fine‐tuning by rounding the weights and biases to the nearest 16‐bit floating‐point number without retraining.

### 2.5 Evaluation

After training, fine‐tuning, and quantization of the model, we evaluated the model performance on several motion estimation tasks. The accuracy of the ${\text{DVF}}_{\text{TEMPEST}}$ was assessed using two metrics: the voxel‐wise EPE compared to a ground‐truth DVF, and the mean and standard deviation of the target registration error (TRE). The mean and standard deviation of the EPE was computed over the entire field‐of‐view, within the body contour, and within the lungs. The body mask was obtained by thresholding the normalized MR image >0.1, selecting the largest connected component, and performing a morphological closing. The lung mask was obtained by thresholding the normalized MR image within the body <0.03, selecting the largest connected component, and performing a morphological closing.

The impact of end‐to‐end training versus serial training was measured by comparing the mean EPE of the two models on the test set over the entire FOV, within the body contour, and within the lungs. Statistical significance (p<0.01)of the difference in mean EPE was established by the Wilcoxon signed‐rank test.

The registration performance was evaluated by applying the DVF to the moving, CS‐reconstructed volume and estimating the similarity between this warped volume $I_{\text{warped}}$ and the static, CS‐reconstructed volume $I_{\text{static}}$. This similarity was computed using the SSIM metric^40^ and the normalized root‐mean‐squared error $\text{NRMSE}=\frac{\sqrt{\text{MSE}(I_{\text{static}},I_{\text{warped}})}}{\rho_{\text{warped}}}$ where $\rho_{\text{warped}}$ is the mean image intensity of $I_{\text{warped}}$.

#### 2.5.1 Respiratory‐resolved volumes

TEMPEST was evaluated on the four‐dimensional respiratory‐resolved test set consisting of five patients. Model output was compared using the EPE metric (μ±σ) against the DVF computed with optical flow computed on CS reconstructions (${\text{DVF}}_{\text{OF,CS}}$). Moreover, we measure registration performance by registering the CS‐reconstructed dynamic volume to the static volume using the ${\text{DVF}}_{\text{TEMPEST}}$. The registration performance was quantified using the SSIM metric and the NRMSE (μ±σ) over the entire FOV, within the body contour and within the lungs.

#### 2.5.2 Digital phantom

TEMPEST was evaluated without retraining on a digital phantom, as this allows for comparison to a ground‐truth DVF. The XCAT digital phantom^41^, ^42^ was simulated with MR contrast with equal voxel size and field‐of‐view size as our training data, as described in Table 1, column “4D MRI.” The phantom was simulated for frame frames with respiratory motion up to 50 mm in the anterior–posterior direction and 100 mm in the feet–head direction. Motion with a magnitude this large is unlikely to occur in patients, but allows us to evaluate TEMPEST in situations with large deformations. We compared ${\text{DVF}}_{\text{TEMPEST}}$ to ground‐truth DVFs (${\text{DVF}}_{\text{GT}}$) provided by the digital phantom, which were postprocessed using the framework by Eiben et al^43^ for improved accuracy. Retrospective undersampling was performed using a GA‐SOS readout for undersampling factors 1, 4, 8, 10, 20, 30, 40, and 50. For every undersampling factor, the quality of ${\text{DVF}}_{\text{TEMPEST}}$ was evaluated using the EPE (μ±σ) compared to ${\text{DVF}}_{\text{GT}}$ over the entire FOV, within the body contour and within the lungs.

#### 2.5.3 Physical phantom

Time‐resolved 3D cine‐MRI of a physical phantom (QUASAR MRI 4D Motion Phantom, Modus QA, Ontario, Canada) was acquired on a 1.5 T hybrid MRI‐Linac (Unity, Elekta AB, Sweden). The phantom consisted of an insert in a water tank and was acquired with and without motion applied to the insert. During the “moving phantom” acquisition, the insert moved according to a sinusoidal trajectory with 1/7 Hz frequency and 20 mm amplitude. The relevant scan parameters are listed in Table 1, column “Moving phantom.” During the “stationary phantom” acquisition, for which the relevant scan parameters are listed in Table 1, column “Stationary phantom,” we tested the sensitivity of the streaking artifacts on the motion estimation performance. The performance of TEMPEST and optical flow were assessed by computing the mean absolute error and Pearson correlation between the ground‐truth phantom motion and the z‐magnitude of ${\text{DVF}}_{\text{TEMPEST}}$ and ${\text{DVF}}_{\text{OF}}$, without retraining TEMPEST.

#### 2.5.4 Fully sampled CT data

To test the generalizability, we evaluated TEMPEST on a publicly accessible 4D respiratory‐resolved CT dataset^44^ without retraining the model. The quality of ${\text{DVF}}_{\text{TEMPEST}}$ was assessed using the EPE metric (μ±σ) within the body contour compared to ${\text{DVF}}_{\text{GT}}$, which was provided by the dataset. Moreover, the registration performance was evaluated using the TRE (μ±σ) using 41 landmarks within the lungs, which were provided by the dataset for every frame.^44^

#### 2.5.5 Real‐time motion estimation

To evaluate the time‐resolved motion estimation performance, we acquired undersampled MRI from two healthy volunteers on an MR‐Linac using a GA‐SOS readout and a golden‐mean radial “kooshball” readout. Both scans were acquired without contrast agent injection and were reconstructed using the NUFFT‐adjoint operator after performing radial view‐sharing between a dynamic and the two adjacent dynamics.^45^ During the kooshball acquisition, a feed‐head spoke was acquired every 25 spokes, which provided a self‐navigation signal in the feed‐head direction.

The relevant scan parameters are listed in Table 1, columns “Volunteer 1” and “Volunteer 2,” respectively.

For volunteer 1, we evaluated TEMPEST performance by comparing magnitude of ${\text{DVF}}_{\text{TEMPEST}}$ in the feet‐head direction (i.e., motion trace) to the self‐navigation signal present in GA‐SOS acquisitions.

For volunteer 2, we evaluated TEMPEST performance by computing the Pearson correlation between the magnitudes of ${\text{DVF}}_{\text{TEMPEST}}$ in the feet‐head direction to the self‐navigation signal obtained from navigation spokes, as no reliable quantification of the true motion is available at this high undersampling factor. The undersampling factor for kooshball MRI was given by $R=\left(M_{x}\cdot M_{y}\cdot \pi /2\right)/N_{sp}$, where $N_{sp}$ is the number of spokes per dynamic and $M_{x}=M_{y}=108$ is the matrix size in the x and y direction.

#### 2.5.6 Time

We measured whether TEMPEST is fast enough for real‐time applications by reporting the time for MR acquisition, image reconstruction, and motion estimation. We measured the model inference time (μ±σ) at fp32 and fp16 resolution over 50 evaluations for static/dynamic volume pairs from the test set with a matrix size of 206×206×77 at full resolution. We considered our approach fast enough for real‐time MRIgRT if the total time ≤500 ms, as suggested by Keall et al.^10^

## 3 RESULTS

Based on the hyperparameter evaluation, we found that α=0.8 and K=3 to be optimal among those that we evaluated. The full results are presented in Supporting Information Figures S1–S3, which can be found in the Supporting Information Document. For the sizes of the convolution kernels, we found that $c_{0}=3,c_{1}=5,\;\text{and}\;c_{2}=3$ to be best performing, where k=0 is the lowest spatial resolution, and k=2 is full spatial resolution, resulting in a model with 859 660 trainable parameters.

We trained two variants of TEMPEST with these hyperparameters: with serial training and with end‐to‐end training. The network operating at the lowest resolution level was trained for 250 epochs in approximately 4 h with a batch size of 8. The network operating at the second resolution level was trained for 150 epochs in approximately 8 h with a batch size of 4, while also performing backpropagation over the lowest resolution level. The network operating the highest resolution level was trained for 125 epochs in approximately 12 h with a batch size of 4, while also performing backpropagation over both models operating at lower resolution levels.

An example of a DVF produced by TEMPEST from undersampled MRI (R=23) is shown in Figures 3(a)–(c). ${\text{DVF}}_{\text{TEMPEST}}$ shows good agreement with ${\text{DVF}}_{\text{CS,OF}}$ (Figures 3d– f). In this particular case, the mean EPE was 2.78 mm. Animated figures of TEMPEST DVFs computed on 4D MRI are provided in Supporting Information Videos S1–3.

The quality of TEMPEST DVFs significantly increased when using end‐to‐end training compared to serial training on our test set, as shown in Figure 4. For example, the average EPE at R=15 reduced from 3.47±0.76 mm using serial training to 2.25±0.70 mm using end‐to‐end training within the body contour (Wilcoxon, p≪0.001). At the same time, the average SSIM increased with ≥6% at R≥15 (Wilcoxon, p≪0.001) when using end‐to‐end training, indicating better registration performance.

We quantized the weights and biases of TEMPEST from full‐precision (fp32) to half‐precision (fp16). Our analysis revealed that weight quantization step has negligible impact on the model performance, increasing the mean EPE with only $3.7\times 10^{-4}$ mm. However, weight quantization reduced the inference time of a static/dynamic volume pair of matrix size 206×206×77 from 81±7.4 to 31±2.9 ms on an NVIDIA V100 GPU, reducing the total latency.

Based on these results, the quantized, end‐to‐end‐trained TEMPEST model has been adopted for further performance evaluation.

### 3.1 4D respiratory‐resolved motion estimation

**FIGURE 5:**
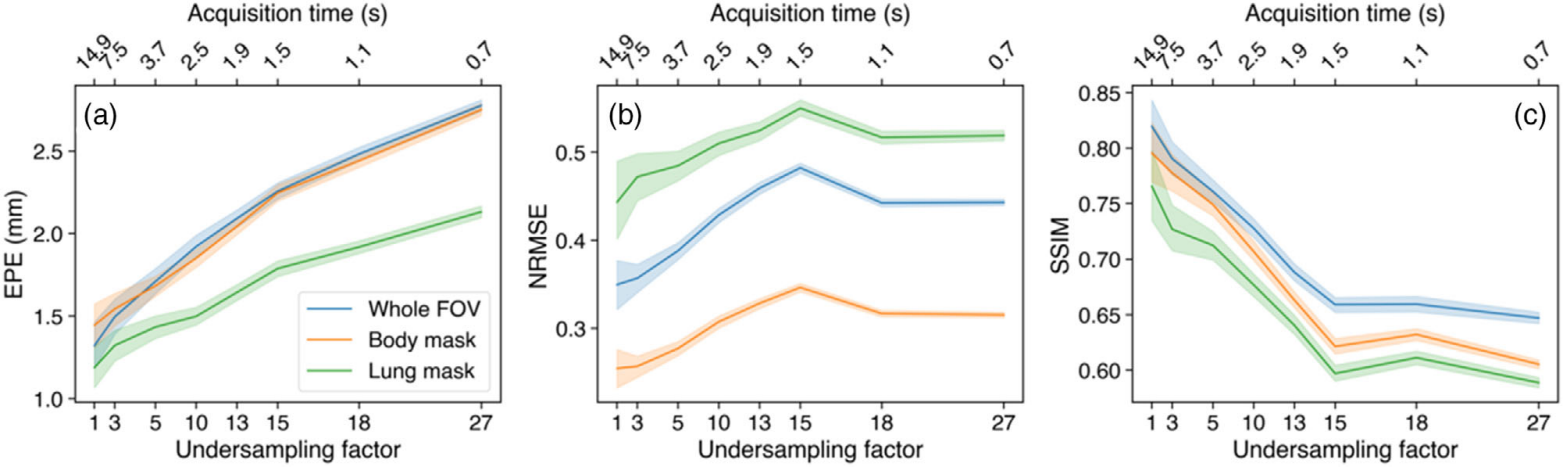
4D evaluation. The EPE (a), NRMSE of compressed sense reconstructions after registration (b), and SSIM of compressed sense reconstructions after registration (c) as a function of the undersampling factor. These metrics were evaluated over the whole FOV (blue), within the body contour (orange), and within the lungs (green). Secondary *x*‐axis shows the approximate acquisition time in seconds using GA‐SOS for that undersampling factor

The performance of TEMPEST on respiratory‐resolved MRI is shown in Figure 5. We found that the EPE remained within 1.9±0.6 mm within the lungs at R=18 compared to ${\text{DVF}}_{\text{CS,OF}}$. The mean NRMSE and mean SSIM plateau at R>15 at approximately 0.51 and 0.63 within the lungs, respectively. Surprisingly, even though TEMPEST has been trained on MRI with an undersampling factor up to R≈7, the mean EPE only moderately increases from 1.5 mm to 1.9 mm at R=18 within the lungs.

### 3.2 Digital phantom

**FIGURE 6:**
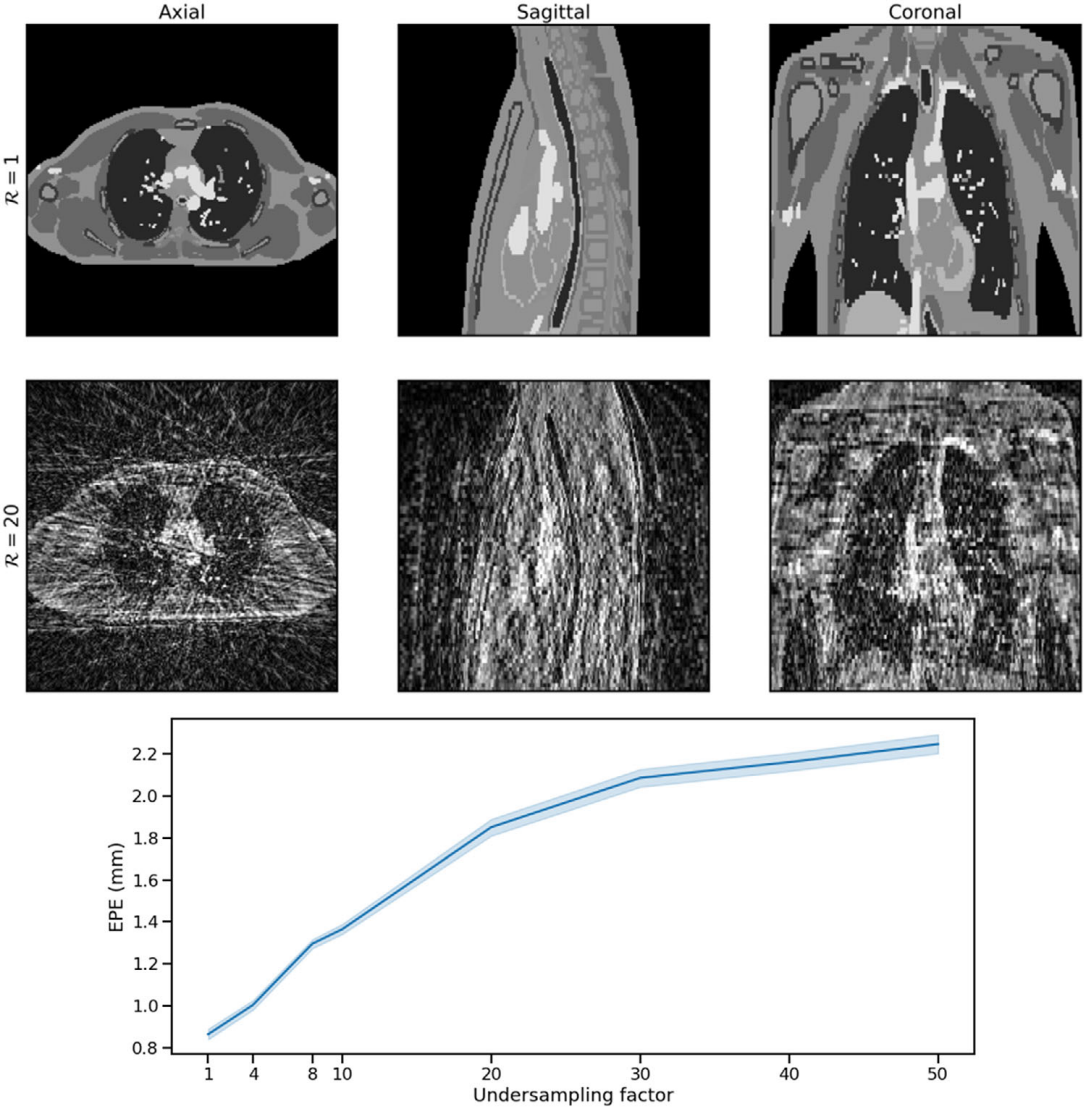
Digital phantom results. An XCAT digital phantom was simulated with up to 50 mm AP motion and 100 mm FH motion. An example of a fully sampled dynamic in exhale is shown in the top row. Volumes were retrospectively undersampled using a GA‐SOS trajectory, for example, R=20, as shown in the middle row. The bottom row shows the EPE (μ±σ) between the model output and the postprocessed ground‐truth XCAT DVF over 100 reconstructions per undersampling factor, using different azimuthal angles and noise

Evaluation of TEMPEST on a digital phantom showed results similar to the respiratory‐resolved test set, as shown in Figure 6. At low undersampling factors, for example, R=4, the mean EPE of ${\text{DVF}}_{\text{TEMPEST}}$ compared to ${\text{DVF}}_{\text{GT}}$ was 0.8±0.12 mm within the body contour. At higher undersampling factors beyond those seen during training, the mean and standard deviation of the EPE increases, yielding a mean EPE of 2.0±0.76 within the body contour at R=30.

### 3.3 Physical phantom

**FIGURE 7:**
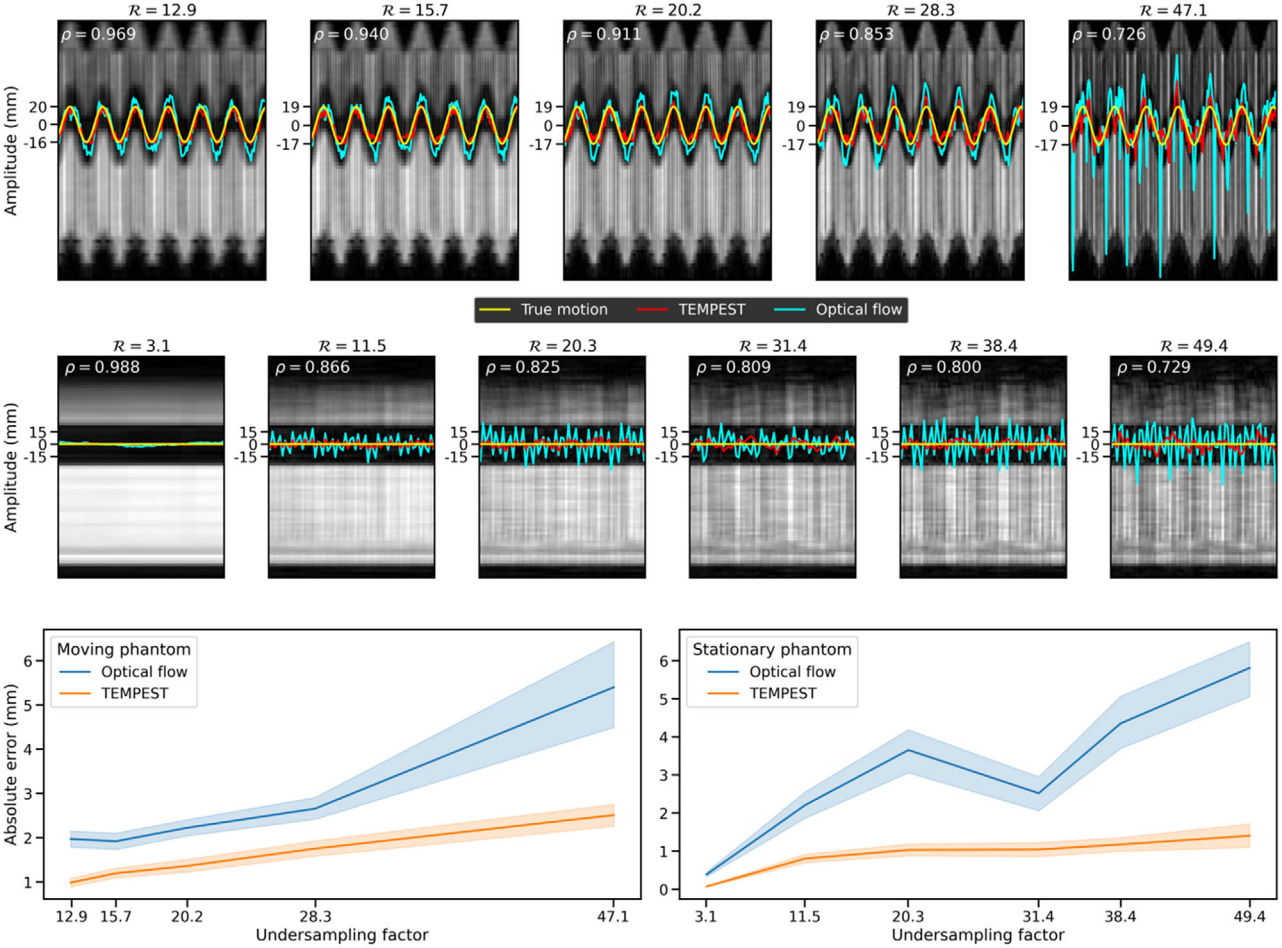
Phantom results. In the background is the progression of a single location along the slice direction over time. In yellow is the ground‐truth motion trace produced by the phantom. In red, the motion trace produced by TEMPEST. In cyan, the motion trace produced by optical flow. This was computed for the phantom in motion (top row) and the stationary phantom (bottom row) for several undersampling factors. The Pearson correlation between the ground‐truth motion and the TEMPEST motion is displayed above the plot as ρ. In the bottom row, the absolute error (μ±σ) is shown as a function of the undersampling factor

In Figure 7, we show the results of the phantom experiments. In the moving phantom experiment (top row), TEMPEST (red) produces motion traces similar to the ground‐truth self‐navigation motion signal (yellow), indicated by the Pearson correlation factor of 0.911 at R=20.2. At R=28.3, TEMPEST estimates motion with an absolute error of 1.75±1.3 mm versus 2.66±1.7 mm for optical flow. For the stationary phantom (middle), optical flow shows significantly more residual motion than TEMPEST at high undersampling factors while there should be no motion. At R=20.3, TEMPEST produces motion traces with an error of 1.03 ± 0.6 mm, whereas optical flow produces an error of 3.65±2.4 mm.

### 3.4 Generalization to CT data

**FIGURE 8:**
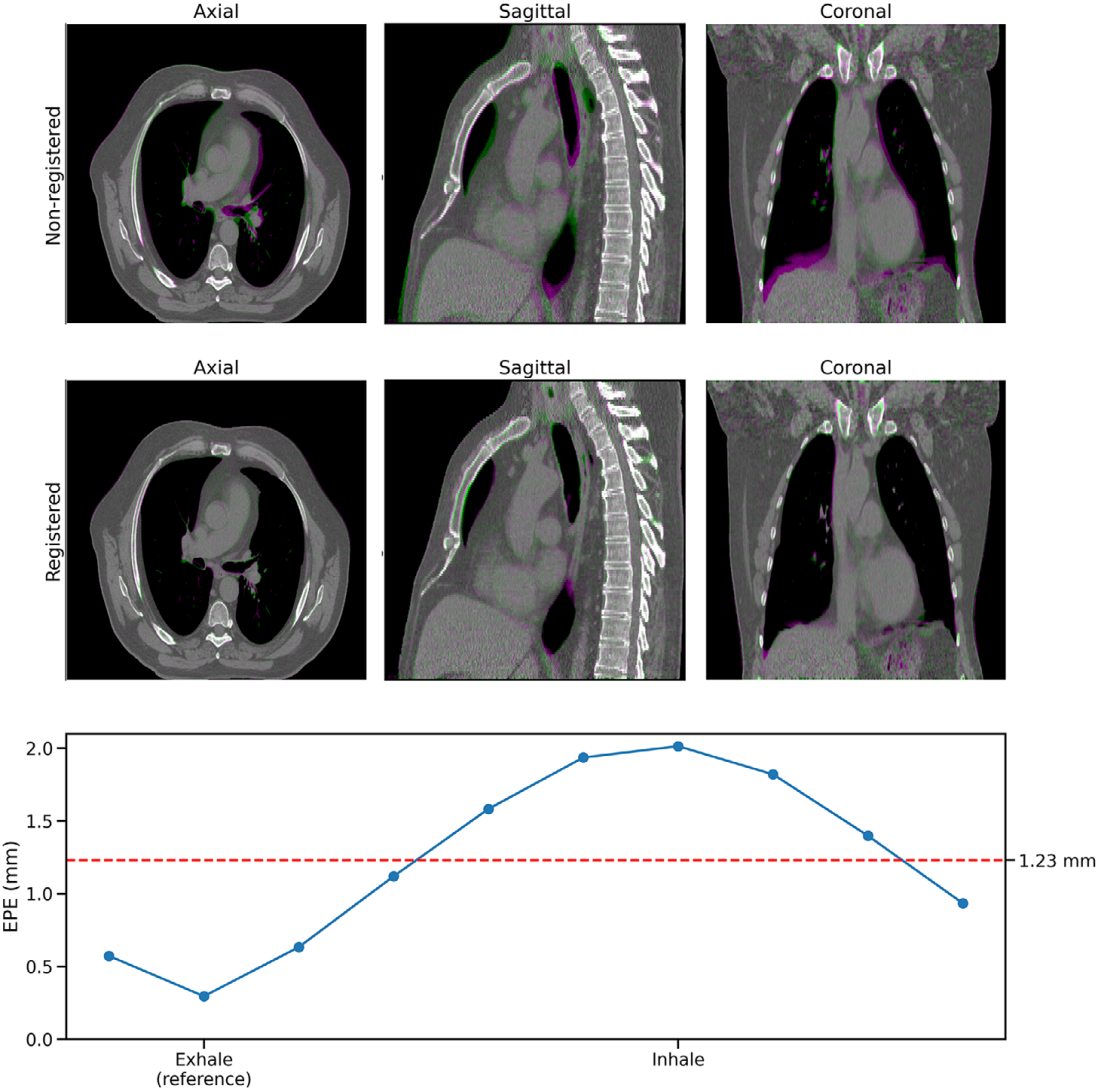
CT results. TEMPEST was evaluated on a fully sampled respiratory‐resolved 4D‐CT dataset. Nonregistered CT shows large differences in image space, especially in the liver dome (top row). In the middle row, it can be seen that TEMPEST is able to register the images with little residual error. On the bottom row, the EPE of TEMPEST compared to the ground‐truth DVF is shown as a function of the respiratory phase. End exhale was the reference phase. The mean EPE was 1.23 mm, shown in the red horizontal line

Surprisingly, when applied to 4D‐CT, TEMPEST estimates motion with low EPE compared to the ground‐truth DVF without retraining the model for this modality. For example, Figure 8 shows that TEMPEST produces DVFs with a mean EPE of 1.23 mm over all respiratory phases, and is able to register CT with little residual motion. When registering images with no motion (e.g., estimating motion from exhale to exhale), the mean EPE was 0.29 mm. The largest mean EPE was observed when registering the inhale CT to exhale, resulting in a mean EPE of 2.01 mm. Registration of the landmarks yielded an average TRE of 1.87±1.65 mm.

### 3.5 Real‐time motion estimation

**FIGURE 9:**
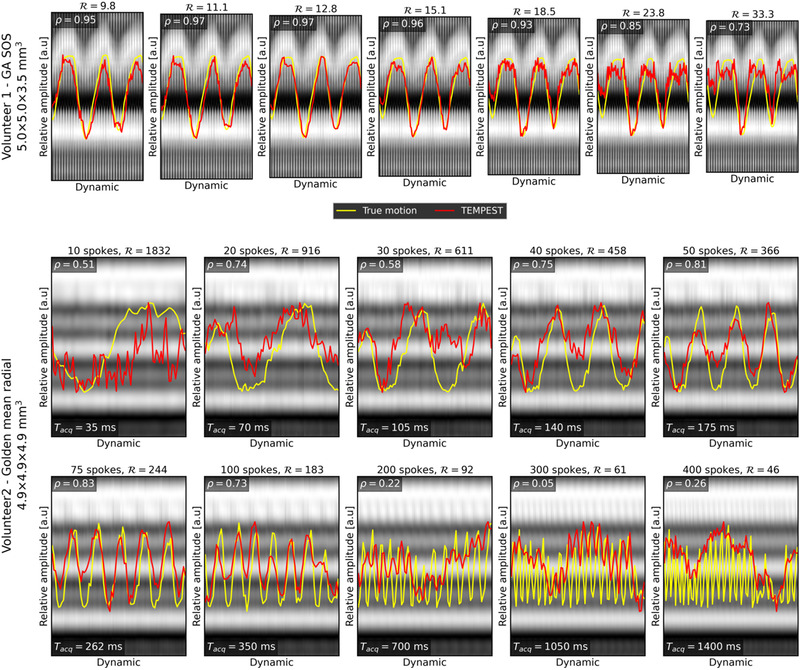
Real‐time results. TEMPEST was evaluated on time‐resolved GA‐SOS volunteer data (top, red line) and compared to the self‐navigation signal (yellow) for multiple undersampling factors. The Pearson correlation between these lines is shown in the same figure. This was also done for another volunteer using a golden‐mean radial kooshball acquisition (middle, bottom). The self‐navigation in a surrogate for the motion in the feet‐head direction in both scans

Evaluation of TEMPEST on time‐resolved MRI is shown in Figure 9. On GA‐SOS k‐space acquired on an MR‐Linac, TEMPEST produces motion similar to the self‐navigation signal, indicated by the Pearson correlation of 0.93 at R=18.5. Animated figures of TEMPEST DVFs computed on time‐resolved GA‐SOS MRI are provided in Supporting Information Video S4–6.

With the golden‐means kooshball readout, we achieve good correlation between the 40 and 75 spokes per dynamic, achieving a Pearson correlation of ∼0.80. Animated figures of TEMPEST DVFs computed on time‐resolved golden‐means kooshball MRI are provided in Supporting Information Video S7–10.

Acquiring one spoke per slice using a GA‐SOS readout takes ∼270 ms for 77 slices. Reconstruction of GA‐SOS k‐space at three resolution levels, where full resolution is 206×206×77 takes about 50 ms per slice using a simple GPU‐based NUFFT algorithm. As TEMPEST takes 30 ms to estimate motion, DVFs can be computed using our methods well within 500 ms, which is the maximum affordable latency for radiotherapy, as suggested by Keall et al.^10^

## 4 DISCUSSION

In this work, we have proposed a DL model called TEMPEST to estimate 3D DVFs from highly undersampled acquisitions to facilitate real‐time MRIgRT applications. In particular, we have presented a multiresolution model that has been trained on respiratory‐resolved MRI that can be used to estimate motion with low latency and high spatiotemporal resolution in time‐resolved MRI. This model is an extension from 2D to 3D of our previously presented approach that estimates motion from undersampled 2D golden‐angle radial acquisitions using NUFFT reconstruction and DL‐based motion estimation.^26^ To the best of our knowledge, this is the first DL model that enables real‐time 3D motion estimation from highly undersampled MRI, with a total latency of less than 500 ms.

We have shown that at R=18 motion was estimated in respiratory‐resolved imaging with less than 2 mm error. The model was validated with various experiments, such as a digital phantom, a physical motion phantom, and 4D respiratory‐resolved CT data. In all these experiments, motion could be accurately estimated with undersampling factors up to R=20. Inference of the model took only ∼30 ms, which is acceptable for MRIgRT.^9^, ^10^ We found that “end‐to‐end” training improved DVF quality compared to “serial” training, decreasing the mean EPE with over 1 mm compared to serial training.

Our experiments with the physical phantom show that TEMPEST is able to accurately estimate motion from data acquired on an MR‐linac compared to the ground truth, even though the model was trained on patient data. Compared to optical flow, TEMPEST DVFs seem to display a greater robustness against the incoherent streaking artifacts present in radially undersampled images. Especially in the stationary phantom experiment optical flow produces a response to aliasing, most notably at higher undersampling factors. Presumably, this is due to the introduced image artifacts present in highly undersampled images.

Even though TEMPEST has been trained on $T_{1}$‐weighted spoiled gradient echo lung MRI, we have demonstrated that our model also performs surprisingly well on different imaging modalities, such as CT, without retraining and yields a TRE of 1.87±1.65 mm. Although these results are promising, state‐of‐the‐art image registration methods or specialized neural networks trained solely on CT images report lower TREs. For example, Marstal et al^46^ showed that Elastix is able to obtain a TRE of 1.58±0.59 mm and Eppenhof et al^47^ obtained a TRE of 1.38±1.24 mm using CNNs. However, these results indicate that the model may generalize well and demonstrates that model has not overfit to a specific imaging contrast. Further experiments are needed to investigate whether TEMPEST also generalizes beyond $T_{1}$‐weighted MRI contrasts, radial MRI, or to different body sites. For example, TEMPEST could be applied to MRI acquired with other non‐Cartesian acquisitions like a stack‐of‐spirals,^48^ golden‐mean cones,^49^ or even Cartesian readouts such as variable‐density Cartesian spirals.^50^ However, this may require retraining as the aliasing changes depending on the sampled trajectory.

For time‐resolved imaging, TEMPEST is able to produce motion traces with high correlation to the self‐navigation signal, as demonstrated in the phantom experiments and the time‐resolved MRI experiments. For GA‐SOS MRI acquired using volunteer 1 at R=18.5, TEMPEST produces DVFs with a motion trace correlating 93% to the self‐navigation signal. For the golden‐mean radial kooshball data, good motion traces can be produce between 40 and 75 spokes per dynamic, corresponding to extreme undersampling factors between R=458 and R=244. We hypothesize for this dataset, this number of spokes provides a good trade‐off between image quality and acquisition latency. With fewer spokes, the undersampling artifacts presumably dominates the motion. With more spokes, longer acquisitions introduce temporal aliasing, as shown in the Supporting Information Videos S7–10. However, radial view‐sharing reduces the effective undersampling factor such that this approach becomes feasible. Moreover, the spatial resolution of 5 mm was significantly larger than the training data. Using larger voxels significantly accelerates the MR acquisition, but reduces image quality as fine details are lost. However, it has been demonstrated that larger voxels have little impact on the estimated optical flow.^51^ With 50 spokes per dynamic, acquisition took ∼175 ms, whereas motion estimation took 30 ms. Our approach thus took 205 ms plus time for image reconstruction, which was within the time budget of 500 ms for real‐time MRIgRT applications, possibly enabling real‐time adaptive MRI‐guided radiotherapy by resolving motion during radiotherapy. For these experiments, we used GPU‐NUFFT implementations that were not fully optimized and assume serial reconstruction of slices. We reckon that highly optimized, parallel NUFFTs can significantly reduce image reconstruction time.

Compared to other works, our method is significantly faster while achieving similar accuracy. For example, Stemkens et al^18^ obtained a 3D motion estimation with an RMSE of 1 mm using a 360 ms 2D acquisition and a few seconds of motion calculation, which is comparable with what we observed. However, this method is not a “full” 3D method but uses multi‐2D cine scans in conjunction with a 4D MRI to obtain 3D motion estimates, possibly limiting the accuracy of the method. Moreover, the computation time of multiple seconds is not fast enough for MRIgRT. Morales et al^24^ proposed an unsupervised DL method to learn 3D DVFs in cardiac imaging with a mean EPE of 2.25 mm. However, their method operated on fully sampled images and needs 9 s of computation for a single DVF, which is not fast enough for MRIgRT. At 10‐fold radial undersampling, we achieve a lower error with an approximately 300 times shorter computation time. Navest et al^52^ used another method to detect motion in MRI. They detected motion from the variance in the noise present in MRI acquisition, achieving fast computation and accurate detection of bulk movement, respiratory motion, cardiac motion, and swallowing. However, although this method may be useful for gated dose delivery, it did not provide absolute motion information per voxel and can therefore currently not be used for real‐time adaptive radiotherapy.

The method we propose is a supervised method and requires ground‐truth DVFs for learning, which could be considered as a limitation given that obtaining high‐quality ground‐truth DVFs for time‐resolved 3D MRI is challenging. We have opted to use optical flow to generate ground‐truth DVFs. Although this is a simple and well‐known motion estimation method, the underlying assumptions of optical flow used to compute DVFs give rise to limited performance in, for example, regions with piecewise constant image intensities.^53^ Using other motion estimation methods, such as Elastix^54^ or demons,^55^ might improve results. Another way to overcome this challenge is by training on synthetic DVFs.^23^ However, the model may learn nonphysiologically plausible DVFs. Also, the training data are then limited to retrospectively undersampled k‐space, which does not suffer from imperfect MRI acquisitions observed in practice. A different way to overcome this challenge is by using an *unsupervised* method.^24^ However, these approaches often use the registration performance as loss metrics,^56^ which may be hindered by for undersampled acquisitions due to image artifacts.

Due to the highly undersampled nature of the time‐resolved MRI experiments and the lack of ground‐truth DVFs, high‐quality validation of TEMPEST is challenging. As severe image artifacts preclude the computation of accurate ground‐truth DVFs, the self‐navigation signal is the most reliable surrogate for ground‐truth motion. However, this is a one‐dimensional motion that only provides relative motion information along one direction, rather than an absolute displacement per voxel along the three axes. Moreover, comparison of global motion information does not allow for motion quality evaluation of specific sites, such as tumors or OARs. In the future, realistic deformable motion phantoms might provide more insight in the motion estimation quality and evaluation on a large patient population could give a better characterization of tumor or OAR motion by using metrics based on anatomical information, such as the Dice score or Hausdorff distance between estimated and ground‐truth segmentations.

Even though TEMPEST was fine‐tuned on highly undersampled images, there is still a response to undersampling artifacts at very high undersampling factors. This could be mitigated by using more sophisticated image reconstruction algorithms, for example, compressed sense or DL‐based image reconstruction. However, as no additional latency is permitted for MRIgRT, these methods are currently not suitable. Although the presented multiresolution approach has proven to produce good results, different DL model architectures incorporating concepts from 2D optical flow, such as cascaded flow inference^37^ and optical flow cost volumes,^36^ have the potential to improve DVF quality at the cost of increased inference times. Another possible cause of the residual undersampling response could be the relatively small training set of 17 patients. Moreover, the estimated hyperparameters might not be optimal as they were optimized for three patients, which might allow for selection of hyperparameters for those three patients instead of all patients. Increasing the number of training samples for hyperparameter estimation and model training might yield improved results at high undersampling factors. An alternative approach may foresee omitting image reconstruction and aim at obtaining DVFs directly from k‐space, as proposed with model‐based methods by Huttinga et al.^57^ However, reconstructing DVFs from k‐space with DL might prove challenging as convolutional operators have strong *local* priors, whereas k‐space contains *global* information.

We believe that DL models are a promising way to facilitate real‐time adaptive MRIgRT, where latency in spatiotemporal resolution has paramount importance. Also, we foresee that TEMPEST could be used for applications that require fast motion estimation or registration of images with artifacts, for example, dose accumulation,^58^ image registration,^54^ or motion‐compensated image reconstruction.^59^, ^60^ In the future, we aim to investigate possibilities to further increase the DVF accuracy at extreme undersampling factors and the spatiotemporal resolution of TEMPEST. TEMPEST could be extended to include temporal information, based on the fact that motion can be represented with spatially and temporally low‐rank models.^19^

## 5 CONCLUSION

We have presented TEMPEST, a DL model that estimates time‐resolved 3D DVFs from undersampled 3D MRI with high spatiotemporal resolution for real‐time adaptive MRI‐guided radiotherapy. To the best of our knowledge, this is the first method to perform real‐time 3D motion estimation from highly undersampled MRI. We have shown that this model can estimate DVFs with high accuracy (<2 mm), low latency, and high spatiotemporal resolution from undersampled radial MRI. TEMPEST estimated DVFs within 200 ms, including MRI acquisition, complying with the requirements for online adaptive MRIgRT. We have evaluated the model performance in‐silico using digital and physical motion phantoms and applied the model to 4D‐CT without retraining. Also, we have shown that TEMPEST can estimate accurate DVFs and achieves good performance in two healthy volunteers.

## CONFLICT OF INTEREST

The authors have no conflicts of interest to disclose.

## Supporting information

Real‐time 3D motion estimation from undersampled MRI using multi‐resolution neural networks

Supporting Information.

Supporting Information.

Supporting Information.

Supporting Information.

Supporting Information.

Supporting Information.

Supporting Information.

Supporting Information.

Supporting Information.

Supporting Information.

## Figures and Tables

**FIGURE A.1:**
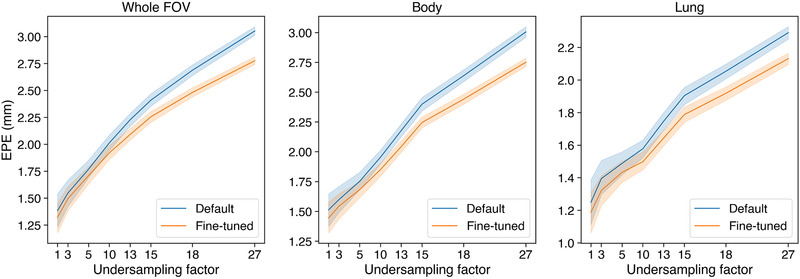
Fine‐tuning results. Two TEMPEST models were fine‐tuned: one using the regular data schedule (blue) and one using our proposed data schedule, featuring highly undersampled images with no motion (orange)

## Data Availability

Research data of this study are not shared.

## Footnotes

1 Code for training and inference will be made available at https://gitlab.com/computational‐imaging‐lab/tempest after acceptance.

## APPENDIX A IMPACT OF FINE‐TUNING

We performed an ablation study to evaluate the impact of fine‐tuning a fully trained TEMPEST on a dataset consisting for 25% of image pairs from the training set up to R=7 *with* motion (i.e., nonzero ground‐truth DVF), and for 75% of image pairs of the training set between 7‐ and 32‐fold undersampling *without* motion (i.e., the ground‐truth DVF is zero everywhere), as described in Subsection 2.4.

We hypothesized that this training schedule improves robustness against severe undersampling artifacts. We tested this hypothesis by fine‐tuning TEMPEST two times:

- Continue training TEMPEST for 100 epochs on the training set, making no changes to the dataset and only presenting MRI undersampled up to R=7.
- Continue training TEMPEST for 100 epochs on the training set using the proposed data schedule.

We evaluated both models after fine‐tuning on the test set using the EPE metric (μ±σ). The EPE was evaluated over the entire FOV, within the body contour, and within the lungs. The results are presented in Figure A.1.

Here, we see that the model fine‐tuned using our data schedule outperforms the default data schedule for every undersampling factor. At low undersampling factors, the impact is small (0.06 mm EPE reduction within the body contour at R=1), but at high undersampling factors the impact increases (0.25 mm EPE reduction within the body contour at R=27).

We conclude that fine‐tuning TEMPEST by exposing the model to extremely undersampled MRI with no motion increases image artifact robustness compared to fine‐tuning using the unmodified data schedule.
