# Supplementary material for: Real‐time 3D motion estimation from undersampled MRI using multi‐resolution neural networks
Source: Med Phys. 2021 Oct 26;48(11):6597–613. doi: 10.1002/mp.15217 (PMC9298075; doi:10.1002/mp.15217)
Supplement: Supplementary file 1 — Real‐time 3D motion estimation from undersampled MRI using multi‐resolution neural networks [file MP-48-6597-s002.doc]

Real-time 3D motion estimation from undersampled MRI using multi-resolution neural networks

M. L. Terpstra^1,2^, M. Maspero^1,2^, T. Bruijnen^1,2^, J.J.C. Verhoeff^1^, J.J.W. Lagendijk^1^, and C.A.T. van den Berg^1,2^

^1^Departement of Radiotherapy, University Medical Center Utrecht, Utrecht, The Netherlands

^2^Computational Imaging Group for MR Diagnostics & Therapy, Center for Image Sciences, University Medical Center Utrecht, Utrecht, The Netherlands

In this supplementary material, we present the results of the hyperparameter optimization and a description of the supplementary information videos.

# Hyperparameter optimization

In this section, we present the results for our experiments to find optimal hyperparameters. In particular, we performed a hyperparameter search to find the optimal value for:

- The number of resolution levels (3 or 4)
- The sizes of the convolution kernels c_k_. The size of the convolution kernels varied depending on the resolution level. For the lower resolution levels, the searched for the optimal value among c_k_ ∊ [3, 5, 7]. For the highest resolution level, c_k_ was fixed to 3 to reduce computational load.
- The value α, weighing the loss function between the EPE, magnitude error and angle error. We searched for α ∊ [0.3, 0.4, 0.5, 0.6, 0.7, 0.8, 0.9]. Here, higher α results in less importance to the EPE term and higher importance of the separated magnitude error and angle error terms.
- The value of β, regularizing the smoothness penalty of optical flow. We searched for β ∊ [0.2, 0.3, 0.4, 0.5, 0.6, 0.7]. Here, higher β results in spatially smoother DVFs.

This resulted in a total of 1188 different model configurations, which were trained on 5 patients and evaluated on 3 patients. For each of the 1188 combination of hyperparameters, a model was trained for 50 epochs on 5 patients with a fixed random seed. We selected the hyperparameters corresponding to the model that achieved the lowest average EPE on three unseen patients.

## Number of resolution levels

##
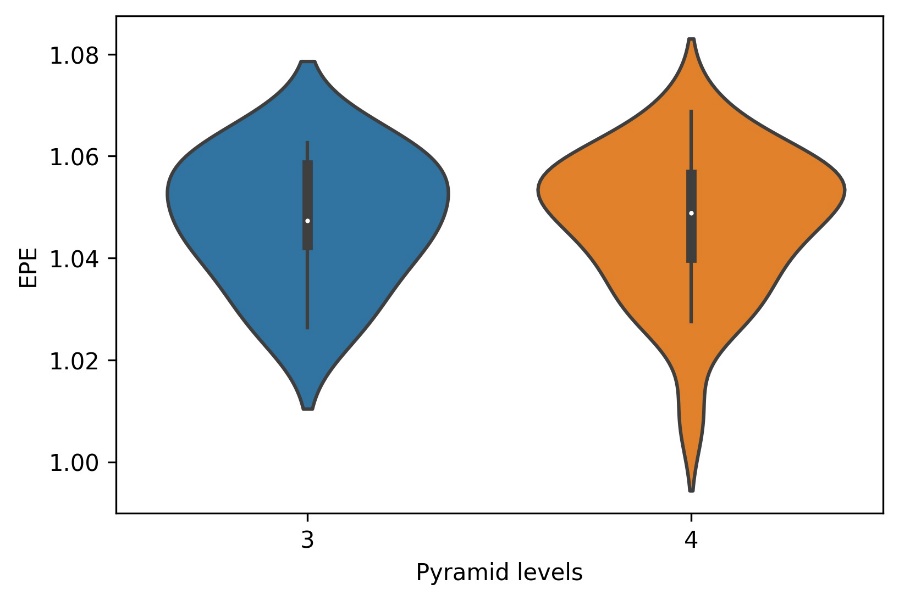

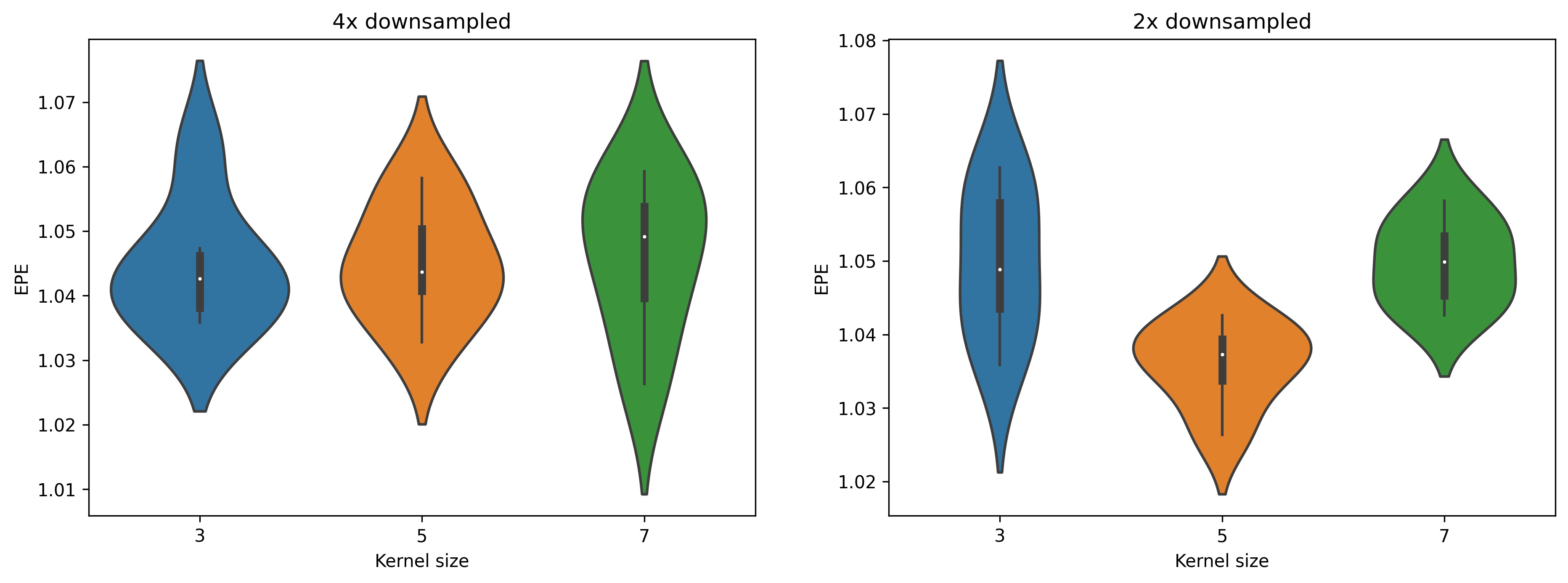
 Sizes of the convolution kernels

**Supporting Information Figure 1**: The EPE as a function of the number of resolution/pyramid levels. Here we see that 3 pyramid levels performs similar to four resolution levels. The mean value for three resolution levels is slightly lower than four resolution levels, but has a shorter tail towards lower EPEs. Therefore, we adopted three resolution levels for our final model.

**Supporting Information Figure 2**: The EPE as a function of the convolution kernel sizes. At 4x spatially downsampled resolution, a kernel size of 3x3x3 shows the lowest mean error, which was adopted as this significantly reduces the number of model parameters. For the network operating at 2x spatially downsampled MRI, we chose 5x5x5 convolution kernels, as this kernel size shows a significantly lower EPE than 3x3x3 and 7x7x7 convolution. For the final resolution level, we adopted 3x3x3 convolution kernels to reduce the computational load.

## The value of α


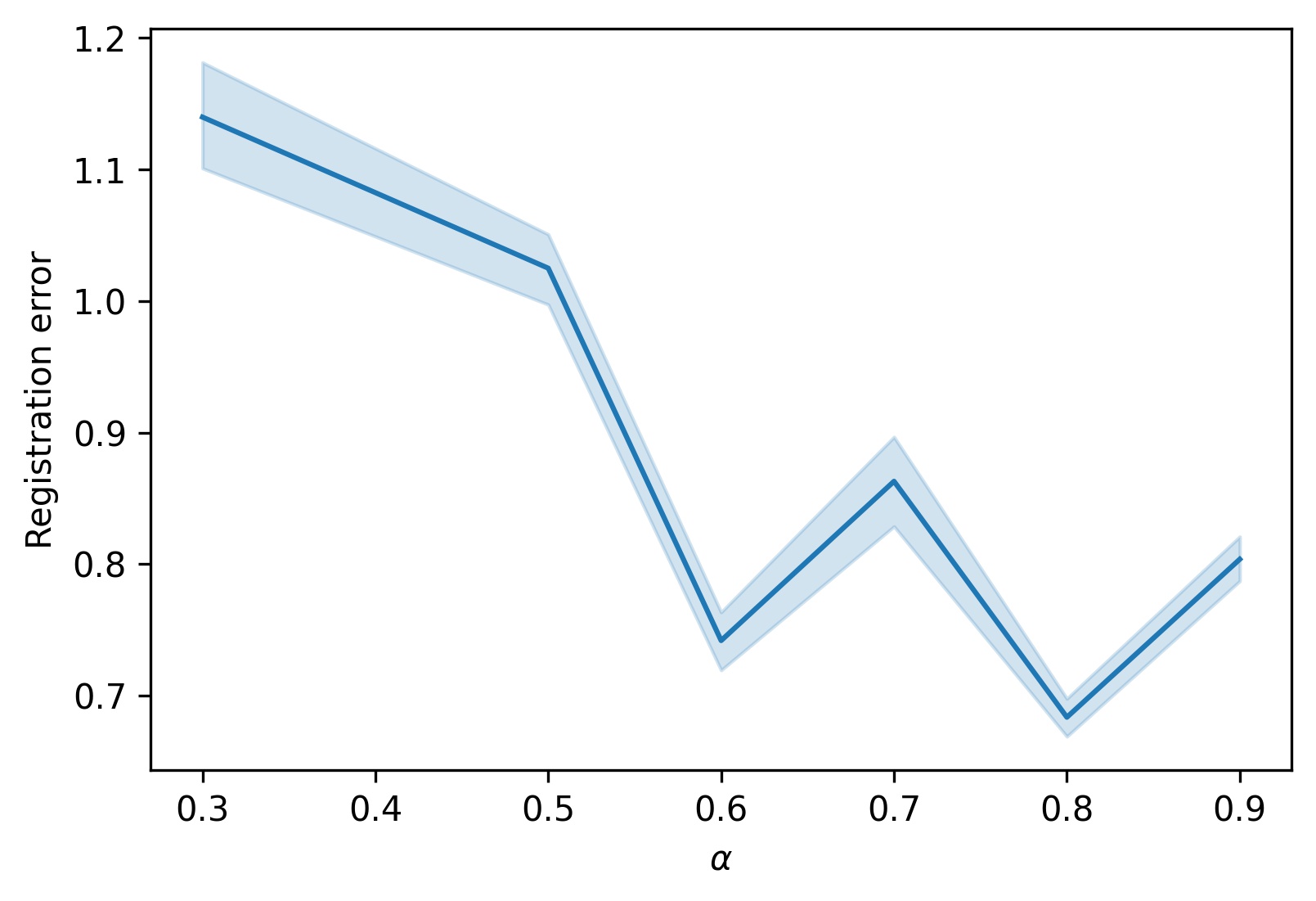


**Supporting Information Figure 3**: The average EPE of the models as a function of α. Here α=0.8 is minimal, so this value was adopted.

## Optical flow


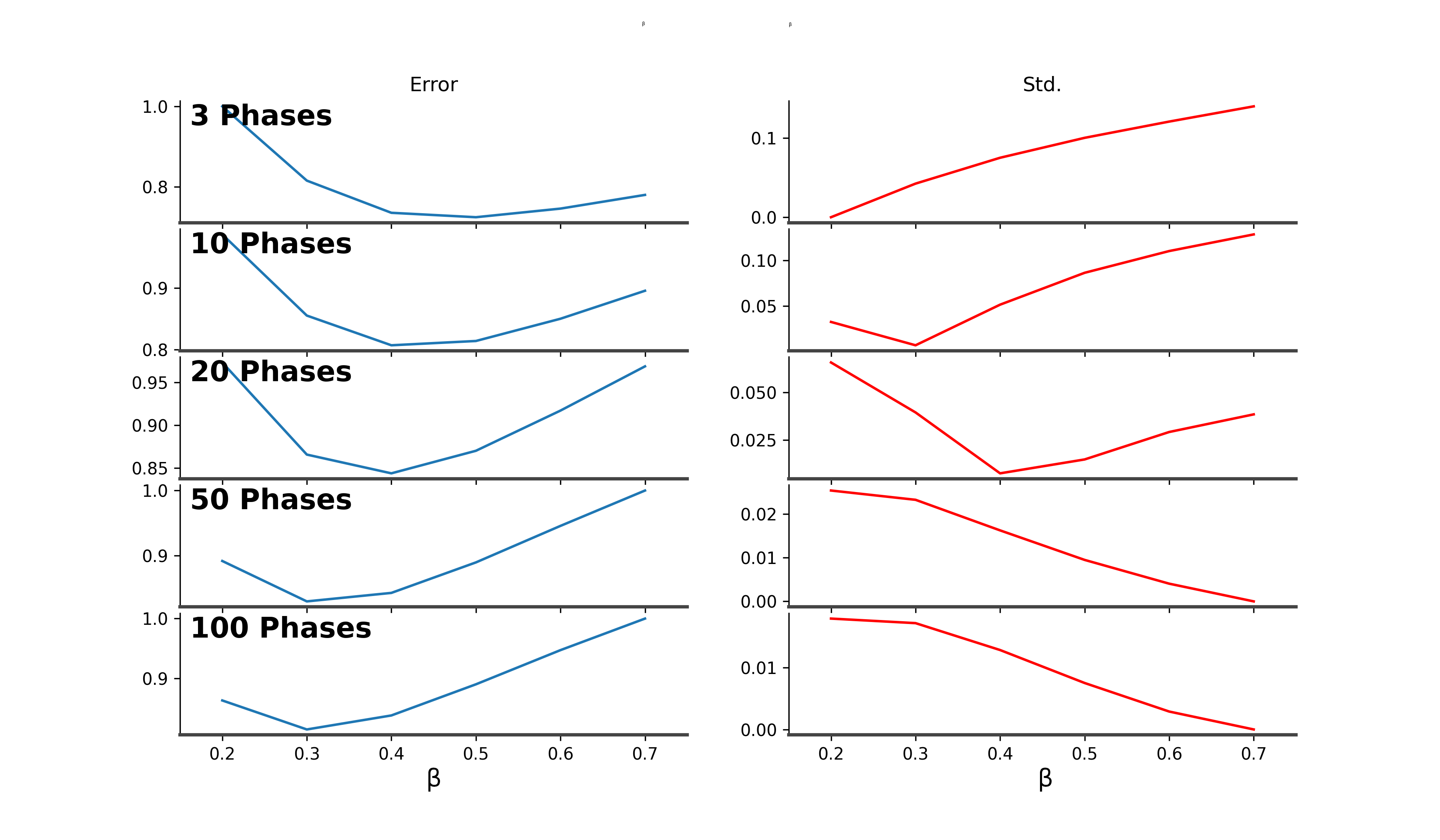


**Supporting Information Figure 4**: The effect of β on the registration error. Left plots show the registration error over a dataset for a value of β. Right plot shows the standard deviation of the registration error. Every row shows the registration error/standard deviation for 4D-MRI with n respiratory phases, i.e. the undersampling factor. We see that for <= 20 respiratory phases, β=0.4 is (near)-optimal. For higher undersampling factors, optical flow favors less smooth DVFs, presumably to resolve streaking artifacts. Therefore, β=0.4 was adopted.

# Description of video files

In this section, we provide extra information for the video material. There are three types of video results: 4D-MRI, time-resolved stack-of-stars MRI, and 3D radial kooshball MRI:

- 4D-MRI
  - **Supporting Information Video S1**: Golden-angle radial stack-of-stars MRI of a patient from the test set was reconstructed with a non uniform fast Fourier transform using 30 respiratory phases, yielding an effective undersampling factor of 8. TEMPEST DVFs on these reconstructions are shown in the top row, while optical flow computed on compressed sense reconstructed MRI is shown in the bottom row.
  - **Supporting Information Video S2**: Golden-angle radial stack-of-stars MRI of a patient from the test set (a different patient than shown in Supporting Information Video S1) was reconstructed with a non uniform fast Fourier transform using 30 respiratory phases, yielding an effective undersampling factor of 8. TEMPEST DVFs on these reconstructions are shown in the top row, while optical flow computed on compressed sense reconstructed MRI is shown in the bottom row.
  - **Supporting Information Video S3**: Golden-angle radial stack-of-stars MRI of a patient from the test set (a different patient than shown in Supporting Information Video S1 and Supporting Information Video S2) was reconstructed with a non uniform fast Fourier transform using 70 respiratory phases, yielding an effective undersampling factor of 18. TEMPEST DVFs on these reconstructions are shown in the top row, while optical flow computed on compressed sense reconstructed MRI is shown in the bottom row.
- Time-resolved golden-angle radial stack-of-stars MRI
  - **Supporting Information Video S4**: Time-resolved golden-angle radial stack-of-stars MRI was acquired using a healthy volunteer on an 1.5T MRI-Linac. MRI was reconstructed using a non uniform fast Fourier transform with 11 spokes per dynamic, yielding an undersampling factor of 15.1. A sliding window with 1 spoke increments was used, resulting in a temporal resolution of ~250 ms.
  - **Supporting Information Video S5**: Time-resolved golden-angle radial stack-of-stars MRI was acquired using a healthy volunteer on an 1.5T MRI-Linac (same volunteer as Supporting Information Video S4). MRI was reconstructed using a non uniform fast Fourier transform with 9 spokes per dynamic, yielding an undersampling factor of 18.5. A sliding window with 1 spoke increments was used, resulting in a temporal resolution of ~250 ms.
  - **Supporting Information Video S6**: Time-resolved golden-angle radial stack-of-stars MRI was acquired using a healthy volunteer on an 1.5T MRI-Linac (same volunteer as Supporting Information Video S4). MRI was reconstructed using a non uniform fast Fourier transform with 7 spokes per dynamic, yielding an undersampling factor of 23.8. A sliding window with 1 spoke increments was used, resulting in a temporal resolution of ~250 ms.
- Time-resolved golden-mean 3D radial “kooshball” MRI
  - **Supporting Information Video S7**: Time-resolved golden-mean 3D radial “kooshball” MRI was acquired using a healthy volunteer on an 1.5T MRI-Linac. MRI was reconstructed using a non uniform fast Fourier transform with 20 spokes per dynamic, yielding an undersampling factor of 916. Radial viewsharing with neighboring dynamics was applied to reduce the undersampling factor. At this undersampling factor, there are too much artifacts to resolve motion
  - **Supporting Information Video S8**: Time-resolved golden-mean 3D radial “kooshball” MRI was acquired using a healthy volunteer on an 1.5T MRI-Linac (same as Supporting Information Video S7). MRI was reconstructed using a non uniform fast Fourier transform with 50 spokes per dynamic, yielding an undersampling factor of 366. Radial viewsharing with neighboring dynamics was applied to reduce the undersampling factor. At this undersampling factor, motion can be resolved fairly well despite the undersampling artifacts.
  - **Supporting Information Video S9**: Time-resolved golden-mean 3D radial “kooshball” MRI was acquired using a healthy volunteer on an 1.5T MRI-Linac (same as Supporting Information Video S7). MRI was reconstructed using a non uniform fast Fourier transform with 100 spokes per dynamic, yielding an undersampling factor of 183. Radial viewsharing with neighboring dynamics was applied to reduce the undersampling factor. At this undersampling factor, image quality is improved but the reconstructed motion quality decreased due to temporal aliasing.
  - **Supporting Information Video S10**: Time-resolved golden-mean 3D radial “kooshball” MRI was acquired using a healthy volunteer on an 1.5T MRI-Linac (same as Supporting Information Video S7). MRI was reconstructed using a non uniform fast Fourier transform with 300 spokes per dynamic, yielding an undersampling factor of 61. Radial viewsharing with neighboring dynamics was applied to reduce the undersampling factor. At this undersampling factor, image quality is significantly improved but hardly any motion is present in the images due to temporal aliasing.
